# Economies of scale and scope in publicly funded biomedical and health research: evidence from the literature

**DOI:** 10.1186/s12961-016-0167-3

**Published:** 2017-02-02

**Authors:** Karla Hernandez-Villafuerte, Jon Sussex, Enora Robin, Sue Guthrie, Steve Wooding

**Affiliations:** 1 Office of Health Economics, London, United Kingdom; 2 RAND Europe, Cambridge, United Kingdom; 3 UCL, Department of Science, Technology and Public Policy, London, United Kingdom

**Keywords:** Economies of scale, Economies of scope, Biomedical and health research

## Abstract

**Background:**

Publicly funded biomedical and health research is expected to achieve the best return possible for taxpayers and for society generally. It is therefore important to know whether such research is more productive if concentrated into a small number of ‘research groups’ or dispersed across many.

**Methods:**

We undertook a systematic rapid evidence assessment focused on the research question: do economies of scale and scope exist in biomedical and health research? In other words, is that research more productive per unit of cost if more of it, or a wider variety of it, is done in one location? We reviewed English language literature without date restriction to the end of 2014. To help us to classify and understand that literature, we first undertook a review of econometric literature discussing models for analysing economies of scale and/or scope in research generally (not limited to biomedical and health research).

**Results:**

We found a large and disparate literature. We reviewed 60 empirical studies of (dis-)economies of scale and/or scope in biomedical and health research, or in categories of research including or overlapping with biomedical and health research. This literature is varied in methods and findings. At the level of universities or research institutes, studies more often point to positive economies of scale than to diseconomies of scale or constant returns to scale in biomedical and health research. However, all three findings exist in the literature, along with inverse U-shaped relationships. At the level of individual research units, laboratories or projects, the numbers of studies are smaller and evidence is mixed. Concerning economies of scope, the literature more often suggests positive economies of scope than diseconomies, but the picture is again mixed. The effect of varying the scope of activities by a research group was less often reported than the effect of scale and the results were more mixed.

**Conclusions:**

The absence of predominant findings for or against the existence of economies of scale or scope implies a continuing need for case by case decisions when distributing research funding, rather than a general policy either to concentrate funding in a few centres or to disperse it across many.

## Background

Funders typically desire to ensure that support is given to high quality research in a range of areas and institutions. Private industry has a strong commercial imperative driving its decisions about where to invest its research and development (R&D) effort and whether to concentrate that in one or a few research centres or to spread it around multiple centres. Public funders have to answer to the people whose funds they are using. It is therefore important to know whether publicly funded biomedical and health research is more productive if concentrated or if dispersed [1, 2].

We wish to know the extent of any economies or diseconomies of scale or scope, and at what levels of aggregation they arise, to inform decisions on research funding allocation. Economies of scale exist when the average cost per unit of a single output is lower the greater the quantity produced in one place, e.g. the cost per MRI scan falls as the proportion of time a scanner is in use increases. Economies of scope are said to exist when undertaking two different activities in the same place leads to greater output per pound spent than undertaking the same two activities separately, e.g. researching diagnostics in the same place as researching medicines. If there are economies of scale and/or scope, then biomedical and health research funding would produce more and/or higher quality outputs if undertaken in centres characterised by a large number of researchers and/or a variety of research activities. Conversely, if there are diseconomies of scale and/or scope, then research funding would be more productive if undertaken in smaller and/or more narrowly focused research centres. If there are neither economies nor diseconomies of scale or scope, then research outputs per pound spent would be unaffected by the quantity and variety of research taking place in any given research centre.

The mechanisms by which economies of scale might arise in biomedical and health research include the ability, as the scale of research activity grows, to make more efficient use of lumpy items of equipment or buildings or of other fixed costs that are location-specific, such as ready access to a pool of specialist support staff performing managerial and administrative functions (finance, legal, and so on). Diseconomies of scale might arise from conflicting pressures to use key equipment or specialist support staff, or from increasing difficulty in coordinating teams of individuals as those teams grow, or simply from increased bureaucracy. Economies of scope can be thought of as the synergies that arise by undertaking different kinds of research side by side. Diseconomies of scope might arise where the synergies are few but the variety leads to a loss of focus in research efforts. The remainder of this paper concentrates on the prior question of whether there is any quantitative evidence of the existence of (dis-)economies of scale and/or scope in biomedical and health research. The studies we have found do not, in general, quantify the mechanisms that may be causing those (dis-)economies.

This paper presents the results of a literature review undertaken to determine the current state of empirical evidence about (dis-)economies of scale or scope in studies of publicly funded research. The review was part of a study undertaken on a grant from the United Kingdom Medical Research Council. The economics of R&D in the for-profit life sciences industry is the subject of substantial literature, the majority of which focuses on the pharmaceutical industry; see in particular the review by Mestre-Ferrandiz, Sussex and Towse [3]. However, to our knowledge, the study we report here is the first review of the literature on the economics of scale and scope in publicly funded biomedical and health research.

We found empirical evidence about economies of scale and/or scope in biomedical and health research to be spread across a disparate literature. Studies varied according to the breadth/specificity of the research areas analysed within biomedical and health research and whether they included other types of research beyond that field, the kind of research organisations focused on (from teams to whole universities), whether the focus was local or national, whether university teaching was analysed as another output alongside research, whether scale or scope or both were investigated, and the economic methodology adopted, as well as different geographical locations and time periods. The literature was not only disparate but also extensive. We have reviewed in full 60 empirical research papers and summarise them here, in addition to 37 economic methodology papers. Given the large number and variety of the papers we found, it has not been feasible within the scope of this project to undertake an analysis of the specific weaknesses and strengths of each paper. We hope to be able to address that in future research. In the remainder of this paper, we focus on what the numerous empirical studies imply about the presence or otherwise of economies of scale and/or scope, and the extent to which the findings vary systematically according to circumstances.

The results of the review should be of interest to biomedical and health research funders and policymakers internationally.

## Methods

We carried out the literature review in two stages; first, reviewing the general econometric literature to ascertain what methods are considered appropriate for the empirical investigation of economies of scale and/or scope in research generally, then reviewing empirical studies of economies of scale and/or scope in biomedical and health research.

### Review of econometric techniques relevant to economies of scale and scope in research

Economies of scale and scope have been investigated in many areas of research activity and a variety of geographical settings and time periods. In December 2014 and January 2015, we undertook a rapid review of the recent econometric literature about estimating economies of scale and scope in research generally. The purpose of this initial review was to understand how economies of scale and scope in research might be analysed empirically, including whether there were any new econometric techniques particularly pertinent to investigating economies of scale and/or scope in research, to help structure our subsequent literature review in the area of biomedical and health research, and to enable us to interpret that literature better.

Given the large volume of econometric literature discussing economies of scale and/or scope in research generally, we focused only on publications from the most recent 6 years, January 2009 to November 2014, as listed in the two main databases of economic literature in the English language: Econlit (through the webpage EBSCOhost) and RePEc (Research Papers in Economics, through the webpage IDEAS). We initially searched on the terms ‘economies’ and ‘research’ and ‘scale’ or ‘scope’, and then filtered first by reviewing article titles, then abstracts. The exclusion criteria are described below. The review of the econometric methods articles was performed by one person and was then checked by two other members of the research team to ensure quality and consistency.

Analysis of economies of scale and scope has traditionally depended on estimation of total cost. This is fairly straightforward when analysing the production of specific goods or services (e.g. computers or insurance), since the total cost can be estimated as the summation of the quantities of required inputs to produce a given unit of output, weighted by the price of each input. However, when analysing production in research, inputs are highly differentiated – for example, labour inputs to research are highly differentiated by the qualifications, skills, knowledge and experience they embody – and corresponding input prices are difficult to obtain. Furthermore, since research is a multi-product process, it is problematic to distinguish how much of which inputs is responsible for the production of a particular research output. Consequently, we excluded from further review those articles that are related to econometric techniques in which the estimation of total costs is based on the multiplication of input prices and input quantities.

Traditional methodologies for estimating economies of scale are based on mono-product processes. However, the process of production in biomedical and health research is inevitably a multi-product process. As mono-product methodologies are inadequate for the analysis of economies of scale and scope in research, we excluded papers that did not consider a multi-product process. We ultimately analysed the full texts of 36 papers from this rapid review.

### Review of literature on economies of scale and scope in biomedical and health research

The findings from the first review helped us to structure the main review of the literature on economies of scale and scope in biomedical and health research, and helped us to interpret that literature. Systematic reviews bring together previous research on a specific question using a systematic, methodical research process, a critical appraisal of the evidence, and synthesis of findings. We undertook a systematic rapid evidence assessment (REA) focused on the research question: do economies of scale and scope exist in biomedical and health research? or, in other words, is it better to support research in the same place rather than spread support across many places? We selected a REA methodology owing to the unexplored nature of the landscape, we were not aware of any previous literature reviews covering these questions, and did not find any subsequently. The REA revealed a large amount of relevant material (as is explained in more detail below). REAs follow the same structure as systematic reviews, and are similarly replicable and transparent, but are less resource intensive. This is achieved by formally constraining the types of research to be searched, the time period, where to find the research, and in what language [4]. To increase the robustness of our search we ‘snowballed’ by inspecting the references in the papers we found. Moreover, we included grey literature as well as peer review journals. The following search terms were used in the first stage of the REA literature review:

- “(econom* OR diseconom*) of scale”
- “(econom* OR diseconom*) of scope”
- “scope (econom* OR diseconom*)”
- “scale (econom* OR diseconom*)”
- “co-location” OR colocation OR “co location”
- “research unit*”
- “research centre*” OR “research center*”
- “centre* of excellence” OR “center* of excellence”
- “team size” OR “group size” OR “unit size” OR “department size” OR “institute size” OR “institution size” OR “lab size” OR “laboratory size”
- “project team” OR “project team dynamics” OR “project team*” OR “large project team*” OR “small project team*”
- “productive efficiency”
- “technical efficiency”
- ((efficienc* OR perform* OR productivity) AND (scale OR scope))

We performed individual searches of each of the previous terms in combination with the following criteria:

- “health* research” OR “medical research” OR “bio* research” OR “life science* research”

For the review of literature on economies of scale and scope in biomedical and health research we used the databases: EconLit, ASC, BSC, Cinhal, PubMed, Scopus, and Web of Science. In this, as in the econometric literature search, we restricted to articles published in English. We excluded papers that focused on private sector R&D only, focused at too high a level, combining multiple distinct research organisations (e.g. research clusters, research/science parks), and ﻿that reviewed a particular centre in isolation or reported a decision to fund a centre.

**Fig. 1:**
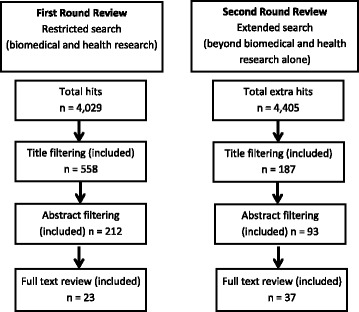
Search results

This first stage yielded 4029 hits, which were reduced to 558 following title filtering and reduced to 212 after filtering by abstract. Further inspection of the full text left 23 papers for inclusion in the review (Fig. 1): 15 were identified by the search strategy directly, to which we added six papers from snowballing from citations in those 15, and two more from personal knowledge of the researchers.

We were concerned that two relevant empirical papers known to one of the authors from previous work had not been found by the search strategy alone. We therefore decided to conduct a second stage literature search relaxing the search criteria to find any empirical papers that covered areas of research that included or overlapped with biomedical and health research, although they might not have focused on economies of scale or scope only in biomedical and health research. To do this, we removed the restriction represented by the health research criteria (“health* research” OR “medical research” OR “bio* research” OR “life science* research”) and relied on our review and exclusion process to exclude articles with no relevance whatsoever to biomedical and health research. This second stage yielded 4405 additional hits, which we reduced to 187 after title filtering, to 93 after abstract filtering, and to 37 after reviewing the full text (Fig. 1).

Thus, taken together, the two stages yielded a total of (23+37=) 60 references that provide information about economies of scale and/or scope in biomedical research. These 60 papers are listed in the References section.

We tested the feasibility of formally and systematically evaluating the papers by drawing on and adapting two existing frameworks as suggested by Gough [5], namely using EPPIs Weight of Evidence Framework and the TAPUPAS dimensions [6]. However, the wide variety of methodologies and approaches found in the literature hinder comparison of the quality of the articles.

Several of the papers analyse higher education institutions as producers of research, postgraduate teaching and undergraduate teaching, rather than focusing on different types of research alone. As they, in part, address the issue of economies of scale in research, including biomedical and health research, these papers are included in our analysis.

## Results and discussion

### Econometric techniques for estimating economies of scale and scope

The literature suggests three main groups of econometric approaches to estimating economies of scale and/or scope. Table 1 shows the 36 articles included in the econometric literature review according to the econometric methodology used. The category called ‘Others’ represents those publications whose methodology cannot be grouped.

**Table 1:** Classification of articles on econometric techniques to estimate economies of scale and/or scope

Econometric technique to estimate economies of scale and/or scope	Articles
Group 1. Through the selection of proxy variables	Arora et al. [7], Brahm & Tarziján [8], De & Nagaraj [11], Horta & Lacy [56], Plotnikova [9], Qin & Buccola [57], Shakina & Barajas [10]
Group 2. Analysis of multi-product cost functions	Agasisti & Johnes [14], Duch-Brown & Parellada-Sabata [18], Fu et al. [58], Johnes & Johnes [15], Johnes & Schwarzenberger [16], Longlong et al. [59], Mamun [19], Martins et al. [20], Sav [17], Sav [13], Worthington & Higgs [60]
Group 3. Analysis of multi-product production functions	Bonaccorsi et al. [27], Cardamone [28], Chavas et al. [21], De Witte et al. [26], Ferrier et al. [24], Ferrier et al. [25], Hadad et al. [29], Podinovski & Førsund [30], Pope & Johnson [23], Ray [61], Schubert [22], Yip et al. [62]
Others	Cho & McCardle [63] (discrete-time infinite-horizon model for a technology-purchasing firm),
	Mayer-Haug et al. [64] (meta-analysis)
	Nemoto & Furumatsu [65] (input distance function approach)
	Olivares & Wetzel [51], Oh et al. [66] (comparison between a parametric production function approach and a nonparametric approach)
	Saal et al. [67] (summary of various methodologies)

Source: Authors’ elaboration

#### Proxy variables

The first group of methodologies analyses economies of scale and/or scope by using proxies for the scale and for the scope of the research group, respectively. Variables such as total number of innovation projects in a research group, total number of projects started by the firm, total number of ongoing projects for the research group at a point in time, and total numbers of students enrolled (in the analysis of scale at higher education institutions), have been used as proxies for research group scale. In some articles, more than one proxy is used, namely one that reflects the scale of the whole research group and another that reflects the scale of the specific unit within the research group, e.g. the size of the research team.

Scope is addressed by using proxies such as thematic concentration of an institution in a specific output, entropy of the uniformity of the distribution of different projects within a research group portfolio, or a Herfindahl index of the diversity of projects in a research group [7–9]. The research group’s Herfindahl index is defined as:

1 $$ H I={\displaystyle {\sum}_{i=1}^N{s}_i^2} $$

and the entropy of the distribution of projects is estimated as:

2 $$ Entropy=-{\displaystyle {\sum}_i^N{s}_i ln\left({s}_i\right)} $$

where *s*_*i*_ is the share of projects in indication *i* as part of the total number of projects (taking projects as a quantitative measure of research group output) undertaken by the research group, and *N* is the number of indications. Thus, in a research group that has equal numbers of projects in two indications, the Herfindahl index equals 0.5^2^+0.5^2^ = 0.5 and the entropy is equal to –(0.5*ln(0.5)+0.5*ln(0.5)) = 0.69. While a research group that has equal numbers of projects in each of three different indications has a Herfindahl index equal to 0.33 and an entropy equal to 1.10. The smaller the Herfindahl index, or the greater the entropy, the greater is the research group’s assumed scope.

After the selection of a proxy to measure the scale or scope, the relationship between the proxy variable and the output level is analysed. For instance:

3 $$ {Y}_i = \alpha +{\beta}_1 ScaleProx{y}_1+{\beta}_2 ScaleProx{y}_2 + {\beta}_3 ScopeProxy+{\displaystyle {\sum}_{l=1}^k{\delta}_l{X}_l+\varepsilon} $$

Where *Y*_*i*_ is the total production of output *i* (i = 0,…,N), *α*, *β*_1_, *β*_2_, *β*_3_ and *δ*_*l*_ are parameters to be estimated, *X*_*l*_ corresponds to a set of independent variables, and *ε* is the error term. Depending on the characteristics of the databases used and the interests of the authors, a variety of standard econometric techniques have been applied in the literature to estimate this type of equation, e.g. ordinary least squares [8, 10], random effects model [11], probit [7], structural [7], and logit models [9].

#### Multi-product cost functions

The methodology of the second group of articles (Table 1) is based on the analysis of the multi-product cost function. The specification of this cost function was originally proposed by Baumol et al. [12]. In particular, the quadratic functional form of the multi-product cost function is estimated. Here, the total cost of producing N different outputs is dependent on the level of production of each of the outputs and on a group of control variables, as follows:

4 $$ T{C}_N=\alpha +{\displaystyle {\sum}_{i=1}^N{\beta}_i{Y}_i}+{\displaystyle {\sum}_{i=1}^N{\displaystyle {\sum}_{j=1}^N{\beta}_{i j}{Y}_i{Y}_j}}+{\displaystyle {\sum}_{l=1}^k{\delta}_l{X}_l+\varepsilon} $$

Where *Y*_*i*_ are the output variables, *TC*_*N*_ is the total cost of producing all outputs, *ε* is again the error term, *X*_*l*_ are control variables, and the other terms are coefficients to be estimated.

In this methodology, two types of economy of scale in a multi-product process are addressed, namely ray economies of scale and product-specific economies of scale. Ray economies of scale are the impact on cost resulting from an increase in the production of all types of output by the same proportion. Product-specific economies of scale are the impact on cost of increasing the production of only one of the outputs while keeping production of all other outputs unchanged. Thus:

5 $$ \mathrm{Ray}\ \mathrm{economy}\ \mathrm{of}\ \mathrm{scale}= T{C}_N/\left[{\displaystyle {\sum}_{i=1}^N MarginalCos{t}_i*{Y}_i}\right] $$

6 $$ \mathrm{Product}\ \mathrm{specific}\ \mathrm{economy}\ \mathrm{of}\ \mathrm{scale} = \left[ T{C}_N- T{C}_{N- i}\right]/\left[ MarginalCos{t}_i*{Y}_i\right] $$

Where *TC*_*N* − *i*_ is the total cost of producing all outputs but *Y*_*i*_.

The ray economy of scale, equation (5), is the ratio between the total costs of producing all outputs together and the sum of the outputs weighted by their marginal costs. If the total cost is higher than the sum of the marginal costs, an increase in the whole level of production across the board will increase the total cost less than proportionally. Therefore, if the resulted value from equation (5) is higher than the unity, there remain some untapped economies of scale.

Product-specific economies of scale are measured through the ratio between the difference in the cost of a production process with and without the output *i* and the production of *Y*_*i*_ alone weighted by its marginal cost (equation (6)). Again, a value higher than unity means that there is an untapped economy of scale in the production of output *Y*_*i*_.

The concept of economies of scope differs from the previous two concepts in that what is important is not so much the size of the research group, but the mix of products. Methodologies that are based on the multi-product cost function analyse economies of scope through the ratio between the total cost of producing output *Y*_*i*_ in one firm and all other outputs separately in another firm (*TC*_*i*_ + *TC*_*N* − *i*_), with the total cost of producing all outputs within one firm (*TC*_*N*_), as shown in the following equation:

7 $$ \mathrm{Economies}\;\mathrm{of}\;\mathrm{scope}=\left[ T{C}_i+ T{C}_{N- i}- T{C}_N\right]/ T{C}_N $$

In this case, values higher than zero indicate that economies of scope exist.

Equations (5), (6) and (7) are based on the estimation of equation (4), which can be done using a variety of econometric techniques. Depending on the data available and the interest of the authors, different econometric techniques have been used, for instance, panel data fixed effects [13], random parameter model [14–16], ordinary least squares [17], Generalized Least Squares [18], random parameter Stochastic Frontier models [19], and maximum likelihood technique [20].

#### Multi-product production functions

In this group of papers (Table 1), the analysis is based on the idea, and estimation, of a production frontier, i.e. the maximum quantities of outputs that can be produced from given inputs in an efficient firm. The methodologies used in this group are mostly non-parametric approaches, the specifications of which vary considerably.

**Fig. 2:**
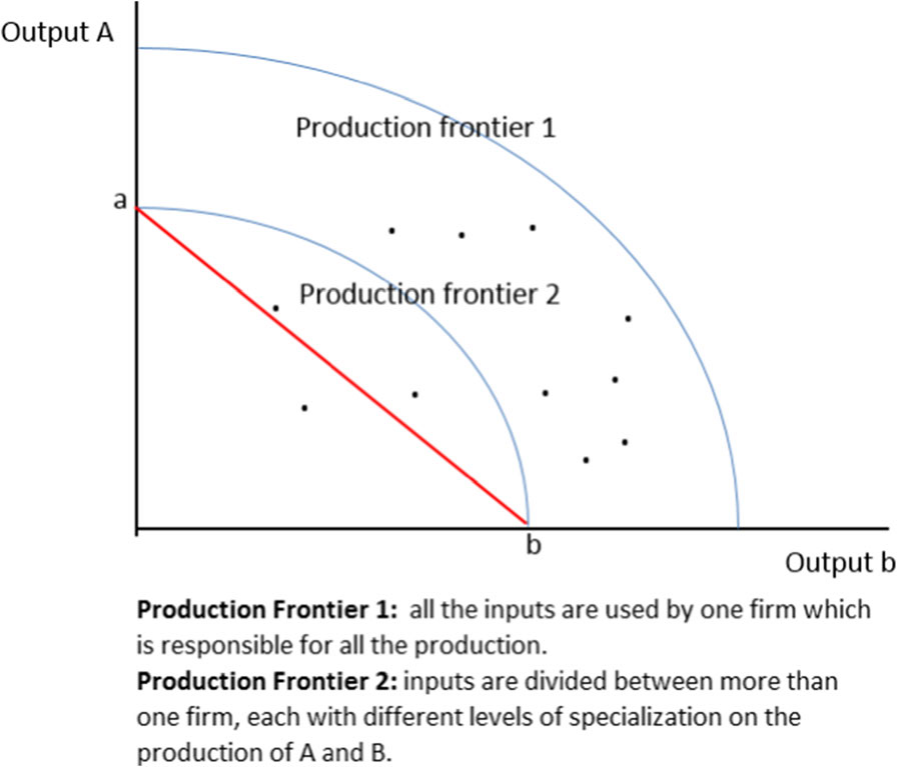
Production frontier with two outputs

One type of approach compares the production frontier of a situation in which a single firm produces all of two different outputs by employing all the available inputs, with the production frontier where the inputs are split across more than one firm, each with different levels of specialisation in the production of output 1 and output 2 [21]; Fig. 2 illustrates this case. The idea is to compare the distance between Production Frontier 1, in which one firm has all the inputs available, and Production Frontier 2, in which the inputs are divided between different firms. Production Frontier 2 is the sum of the production of each firm. In Fig. 2, this hypothetical case shows an example where economies of scale exist for all mixes of the two outputs A and B – the single firm can out-produce multiple firms using the same total of inputs whatever ratio of output A to output B is desired. The shape of the curve in this example implies also that there are economies of scope, namely multiple firms where each firm produces either output A or output B, but not both, could only achieve a combined output along the line AB, which is always below Production Frontier 2 when non-zero quantities of both products are to be produced.

**Fig. 3:**
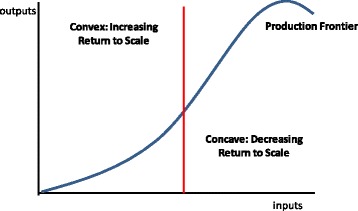
Curvature of the production function

Another approach related to the production frontier analyses the curvature of the production function [22]. Figure 3 shows a production function in which, at lower levels of production, the firm faces increasing returns to scale (the convex part of the curve) and, at higher levels, it faces decreasing return to scale (the concave part of the curve).

There are a variety of methodologies for determining the curvature of the production function for different groups of firms, including a Free Disposal Hull [22, 23], Free Coordination Hull [24, 25], a ‘benefit-of-the-doubt’ approach [26], g or a Data Envelopment Analysis (DEA) [21–23, 27–30]. Curves can be entirely concave, or convex, or constant, or a combination of all three as shown in Fig. 3. In addition, the position of each firm along the curve is determined, such that the firm could be located in the concave or the convex part of the production frontier. Based on both the form of the curve and the position of the firm, it is possible to conclude whether the firm is benefitting from economies of scale.

### Evidence about economies of scale and scope in biomedical and health research

In principle, economies of scale and/or scope in biomedical and health research could come from two main sources. They could result from infrastructure, such as expensive research equipment or costly-to-acquire skills or support services (legal, financial, managerial, administration) that can be provided more cost effectively where a large group of people are making use of that infrastructure and are in the same location. Or they could result from the interactions between researchers, enabling them to work together better and draw on advice from colleagues. Diseconomies of scale and/or scope might result from growing difficulties of coordination when research groups employ larger numbers of people or wider varieties of different kinds of researchers (with different expertise and interests and ways of working). The quantitative analyses we found did not attempt to identify the relative importance of the possible sources of any economies or diseconomies that were detected.

We found 60 papers from the literature review discussing, in at least part of their content, whether there exist economies of scale, or economies of scope, or both, in publicly funded biomedical and health research. Table 2 shows which of the 60 papers concerned economies of scale, which concerned economies of scope, and which concerned both scale and scope. Seven of the 60 papers included discussion of factors affecting the productivity of biomedical and health research, particularly the effect of co-location of researchers, but contained no empirical analysis of economies of scale or scope (last row of Table 2). Of the other 53 papers, 31 look only at economies of scale, two look only at economies of scope, and 20 cover both scale and scope (Table 2). Thus, scale was frequently analysed independently of scope, but scope was seldom analysed in isolation. Methodological approaches are for the most part quantitative, with only a few cases of qualitative, literature reviews and mixed methods studies (Table 2).

**Table 2:** Articles included in the literature review

	Quantitative	Qualitative/Literature Reviews	Mixed Methods
Analyses of economies of scale only	Adams & Griliches [39], Bauer et al. [34], Bonaccorsi & Daraio [68], Bonaccorsi & Daraio [38], Bordons & Zulueta [69], Bordons et al. [70], Cohen [71], Cohen [72], Gomes et al. [73], Heale et al. [35], Hoare [43], Johnes & Johnes [74], Kenna & Berche [33], Kenna & Berche [44], King [50], Kretschmer [32], Mamun [75], Nag et al. [76], Rey-Rocha et al. [77], Sav [36], Schubert [22], Stankiewicz [78]	Cohen [79], Heinze et al. [80], Johnston [81], Rhoten [40], Stokols et al. [82] von Tunzelmann et al. [85] (This is a pure theoretical analysis)	Adams et al. [83], Carayol & Matt [84] Lowry et al. [86]
Analyses of economies of scope only	Cherchye et al. [54], Glass et al. [55]		
Analyses of both economies of scale and economies of scope	Agasisti & Johnes [14], Chavas et al. [21], Cohn et al. [46], de Groot et al. [48], Dundar & Lewis [52], Foltz et al. [41], Glass et al. [45], Hinze et al. [53], Johnes & Johnes [15], Johnes & Salas-Velasco [87], Johnes & Schwarzenberger [16], Laband & Lentz [47], Mamun [19], Olivares & Wetzel [51], Olson [88], Sav [37], Seglen & Aksnes [89], Spanos & Vonortas [31], Wolszczak-Derlacz & Parteka [49]		Vonortas et al. [90]
Analyses of topics closely related to economies of scale or scope (e.g. co-location)	Abramovsky et al. [91] (effect of co-location, R&D labs and university)	Atkinson et al. [92] (change in scientific collaboration along time)	Beise & Stahl [93] (effect of co-location (private and public))
	Antonio-García et al. [94] (analysed the effect of size through the satisfaction with resources)	Bos et al. [95] (co-location of the team members)	
		Chin-Tsai & Chang-Tzu [96] (how research performance can be assessed)	
		Coen et al. [97] (integrating the tangible and intangible structures that underlie research centre functioning (co-location))	

Source: Authors’ elaboration

In the following sections we set out the findings of these papers in more detail, considering in turn economies of scale and then economies of scope.

### Economies of scale in biomedical and health research

The results of the 51 papers evaluating economies or diseconomies of scale in biomedical and health research or in other types of research that include or overlap with biomedical and health research (listed in rows 1 and 3 of Table 2), are far from uniform, although positive economies of scale are found more often than diseconomies of scale. Table 3 summarises the findings from these 51 papers. Forty-two of the 51 papers offer in essence a single overall conclusion about (dis-)economies of scale in biomedical and health research (or offered no clear conclusion on this point). The other nine of the 51 studies were designed to be able to identify (dis-)economies of scale in a number of separate situations and all of them found different results according to the situation of interest.

**Table 3:** Numbers of papers finding (dis-)economies of scale in biomedical and health research – by type of research group whose productivity is being analysed

	Individual level (Principal Investigator)	Research Team^a^	Department^b^	University/Research Institute	Other	Total
Diminishing returns to scale/Diseconomies of scale	2	3	1	3	–	9
Increasing returns to scale/Economies of scale	1	3	2	9	–	15
Constant returns to scale	1	3	1	–	2	7
Inverse U-shape	–	2	–	1	–	3
Not clear	2	1	3	–	2	8
Sub-total	6	12	7	13	4	42
Articles that suggest both diseconomies and economies of scale						
Multi-product cost functions applied	–	–	–	4	–	4
Other methodology applied	–	1	–	4	–	5
Total	6	13	7	21	4	51

^a^Research Team = Sub-departmental laboratory/research unit

^b^University department or research centre inside a bigger organisation. A department is typically composed of a number of research teams

There is no dominant overall conclusion; both economies and diseconomies of scale are reported in the literature. Rather more papers report positive economies of scale (15/42) than report diseconomies (9/42) or constant returns to scale (7/42). Three papers find that, while there are positive economies initially as scale increases, these eventually turn to diseconomies after a certain scale is reached. Spanos and Vonortas [31] detect economies of scale, measured as the number of partners in European research projects, up to 25 such partners but diseconomies of scale thereafter. While Kretschmer [32] found that diseconomies might set in for research group sizes above 6–12 members, Kenna and Berche [33] suggested the turning point might be around 41 group members for medical sciences, 21 for biology and 11 for economics and statistics.

The empirical literature includes studies that focus on a variety of levels of aggregation of research groups, from individual research projects under a principal investigator to whole universities or large research institutes. In Table 3, the results are broken down according to the types of ‘research group’ studied. We use the term ‘research group’ to apply to any team of people working together on research, or to an agglomeration of such teams. We have defined four ‘research group’ types according to the type of unit whose productivity is being analysed in a study. For instance, those papers classified under ‘University/Research Institute’ are analysing differences in productivity between universities and/or research institutes. Productivity might be measured, for example, as the total number of publications from each university/institute in a particular period of time, while the scale measure could be the average size of the research teams within the university/institute. Similarly, in the case of the ‘Individual level (principal investigator)’ category, those studies are measuring the productivity of a particular principal investigator while the scale measure could be the size of their research team.

Among the 51 papers studying economies of scale, six present analyses in which the unit explored is the researcher (Table 3). The number of studies in this category is small and reveals a spread of findings across all of decreasing, constant or increasing returns to scale. In these six studies, neither multi-product cost functions nor multi-product production functions were used. Bauer et al. [34] predict the number of publications per researcher as a function of academic status, sex, department size, and quota of senior researchers, and they predict the number of citations by publication count, status, department size, and quota of senior researchers. Similarly, Heale et al. [35], using information on publications and funding per individual principal investigator, analyse the determinants of scientific knowledge output in biomedical research. They find diminishing returns in relation to the amount of funding available and that a reasonably stable network of external collaborators is key for high productivity.

The 13 studies focused at the ‘Research Team’ and the seven studies focused at the ‘Department’ (sub-university/institute) level reveal a similar spread of results across decreasing, constant and increasing returns to scale (Table 3).

Whole universities or research institutes were the focus in 21 of the 51 studies looking at economies of scale. At that level, nine out of 21 papers show positive economies of scale in research, compared with just three finding diseconomies of scale. For instance, Sav [36], by applying DEA, found that “*productivity regress was accompanied by decreasing returns to scale that prevailed among more than half of the USA universities*”. Nine other studies focused at the university/research institute level find a mixed picture with both increasing and decreasing returns evident in different circumstances.

We focus now specifically on the nine articles studying economies of scale that suggest the existence of both economies and diseconomies of scale, depending on the specific context (Table 3). Four of these nine articles used a multi-product cost function approach and estimated both ray economies of scale and product-specific economies of scale [15, 16, 19, 37]. Johnes and Schwarzenberger [16] used panel data that allowed the estimation of a random parameter stochastic frontier model, meaning that parameters could vary across institutions. Based on this methodology the authors found product-specific economies of scale for research in German universities (i.e. where research activity increases but teaching activity remains unchanged the average cost of the research falls) but minimal ray diseconomies of scale (i.e. when research and teaching increase by the same proportion, average costs of both rise slightly).

Two of the nine articles use DEA analysis [21, 38]. In these cases, for each university/institute analysed, a different measure of economies of scale is estimated, and some of the institutions show diseconomies while others show economies of scale. For instance, of 52 American universities analysed by Chavas et al. [21], 69% (36/52) showed economies of scale and 31% (16/52) diseconomies. The findings of Bonaccorsi and Daraio [38] suggest diseconomies of scale in the French university system and a U-shape pattern (diseconomies in smaller and economies in bigger research institutes) in the case of Italian universities.

Two other articles from these nine applied different proxy variables for scale and estimated more than one equation, and found different views of (dis-)economies of scale according to which proxy for research output was used. For instance, Adams and Griliches [39] found diseconomies of scale in the production of research publications but positive economies of scale in citations.

Finally, the qualitative analysis by Rhoten [40] indicates that an increase in centre size from small to medium could increase the number of information sharing ties but not knowledge creating ties, while an increase from medium to large does not appear to increase either of the two and so would not imply economies of scale over that range.

Taken as a whole across the group of 51 articles looking at economies of scale, number of publications was the most common proxy measure of research output used – in 26 of the 51 papers – reflecting the comparatively ready availability of publication counts. Patents were used as an output measure in just two papers [21, 41, 42]. Out of the 26 articles based on publication counts, two included international publications and 10 consider only publications in indexes of refereed journals. Moreover, 11 of the 26 articles took into account the quality of the publications through the numbers of citations.

An additional proxy measure of research output used in the literature is the quality of research, which applied in four of the 51 articles and was measured using results from the United Kingdom’s Research Assessment Exercise (RAE). The RAE classified university research teams into five quality levels by means of peer review [33, 43–45].

Numbers of students (postgraduate and undergraduate usually counted separately) were also quite frequently used as output of the work of a university or department, alongside research. For instance, Mamun [19] includes as output measures the full-time equivalent undergraduate students and the full-time equivalent masters (MPhil) and PhD students. He also included the research expenditure level as an approximation of the research output. In this case, ray economies of scale were found for the totality of outputs while research and undergraduate teaching show product-specific diseconomies of scale. Postgraduate student numbers are arguably a weak proxy for research output. They may be correlated with research but that correlation might be negative or positive – negative because time spent teaching students is time not spent researching and positive because larger universities, viewed as multi-product firms, may produce both more teaching and more research. Numbers of PhD students may alternatively be seen as an indicator of the scale of inputs to the research process, rather than of the outputs from it. So the use of student numbers as an output is problematic when trying to assess (dis-)economies of scale, or scope, in research.

The extent of grants and other external research funding obtained was used as a proxy for the scale of research output in 11 of the 51 papers. From the perspective of the recipient research group, the ability to win external funding is an indicator of success, but that funding is an input to the research process rather than an output from it when viewed from a societal perspective. When comparing two research groups, A and B, if A wins more research funding than B, we know that it is using more inputs than B but we do not know if that greater funding is being turned into proportionally more or less research output unless we have another proxy measure for output.

#### Results by type of model used

As shown in Table 2 (second and fourth columns), of the 51 articles found that studied economies of scale (either or alone or along with economies of scope), 45 applied a quantitative (41) or mixed (4) methodology for the analysis of economies of scale. We have classified them according to the three main types of models for assessing economies of scale and/or scope found in our review of the general econometric literature, and found 20 articles did so through selection of proxy variables, 13 through multi-product cost functions, 7 through multi-product production functions, and 5 where the method was unclear or other quantitative approaches had been used.

Among the 20 papers based on the proxy variables approach, the findings were varied – two found positive economies of scale, four found diseconomies of scale, five found constant returns to scale, three found evidence of both economies and diseconomies of scale, and three cases showed no clear results. The results of the remaining three cases indicate an inverse U-shaped relationship between scale and productivity of research – as research group size increases, there are positive economies of scale initially, but eventually they cease and further increases in scale are then associated with diseconomies. For example, Spanos and Vonortas [31] found an inverse U-shape between number of partners and networking impacts and a U-shaped effect of budget on goal achievement. They measure networking impacts thorough a construct that reflects strengthening links with other research organisations/businesses and dissemination of research results, while goal achievement is measured through three Likert-type scales of the degree to which the project achieved its scientific, technical and commercial objectives. Spanos and Vonortas [31] found that, with a budget around €1,795,000, the expected value of goal achievement reaches its minimum level, and that networking impacts achieve a maximum point around 24.5 partners. They conclude that the effect of the scale is curvilinear, meaning that, at high levels of scale, the positive returns will begin to diminish. Kenna and Berche [44] find an inverse U-shape between research quality (measured through the RAE) and the number of researchers in the group. They identify two critical masses, a lower limit, which is the minimum number of researchers needed to ensure stability in the team, and an upper limit after which a greater number of members increasingly obstructs meaningful communication and negatively affects the quality of research. Kenna and Berche [44] suggest that these upper and lower limits depend on the field of research; for instance, they found an upper limit for the number of individuals in a research group equal to 41 (±8) for medical science, 21(±4) for biology and 11 (±3) for economics and statistics.

Additionally, 13 articles are based on analysis of multi-product cost functions. Two of these studies found diseconomies of scale and seven found positive economies of scale. It is worth mentioning that the results presented by Cohn et al. [46] have been classified as evidence of positive economies of scale overall, even though, for private universities, the product-specific indicator shows diseconomies of scale in research. This is because the results for the more numerous (and larger on average) public universities show product-specific economies of scale and, moreover, global (ray) economies of scale were found in both public and private universities [46].

The other four of the 13 multi-product cost function articles show a clear difference between the ray economies of scale indicator and the product-specific indicators. Three of them suggest the existence of global (ray) diseconomies of scale together with positive product-specific economies of scale. However, Mamun [19] finds the opposite in the case of public universities in a low-income economy, Bangladesh, namely, that there are positive global (ray) economies of scale but product-specific diseconomies of scale for the output ‘research’. However, as research output is measured by research expenditure (which is a measure of inputs, when seen from a societal perspective), it is difficult to interpret this result.

It is also worth noting that all of the 13 multi-product cost function studies use numbers of students (undergraduate and postgraduate students separately) alongside a measure of research as the outputs of universities/institutes of higher education. Eight out of the 13 articles use ‘grants and other research funding/research expenditures’ as a measure of research output and four use the number of publications. For instance, Laband and Lentz [47] based the estimation of the multi-cost product function on three outputs, (1) the total sum of the federal, state, local and private research grants in millions of dollars, (2) the total number of undergraduate students enrolled in the institution, and (3) the total number of postgraduate students. They found that private as well as public universities are characterised by ray economies of scale and product-specific economies of scale in research and undergraduate education. Patents are used as a research output measure by Foltz et al. [41] and the quality of the research (as measured by the RAE) is used as an output measure by Glass et al. [45].

An important part of multi-product cost function estimation is the choice of control variables used. A common practice is to include academic wages (e.g. average faculty salary) as a proxy for the level of input prices [19, 37, 41, 46, 47]. Likewise, and given the particularly high costs of a medical department, some studies include a dummy variable for those universities or institutions that include a medical school [14, 37, 41, 48].

Regarding the seven articles that were based on analysis of multi-product production functions, five use DEA [21, 22, 36, 38, 49]. A particular case in this group is the study conducted by Wolszczak-Derlacz and Parteka [49], who analysed 259 European universities. They estimated first the efficiency levels of the universities using DEA and then, in a second step, they regressed these efficiency levels on a group of covariates. Among the covariates they included the number of different faculties. Their findings suggest that a university with a higher number of different faculties is more efficient. They consider this result as a possible signal of both economies of scope and economies of scale. Another example is the study by Chavas et al. [21], who broke down the economies of scope effects into three different parts (complementary, convexity and economies of scale effects). They found that the scale effect is particularly important for the small United States research universities. Only one [22] out of the five articles that applied DEA has a unit of analysis different from the university. He analysed German research groups from three scientific fields and found that the efficiency curve estimated by DEA shows increasing returns to scale and constant returns to scale. The two articles that do not use DEA are King [50], who applied a multi-input multi-output model, and Olivares and Wetzel [51], who used an input distance function approach.

#### Types of biomedical and health research

It might be expected that the likelihood of economies or diseconomies of scale might differ according to the particular type of biomedical and health research that is being conducted. However, the studies we found provide no basis for drawing conclusions about different sub-headings of research within ‘biomedical and health’ such as basic research versus clinical research, or pharmaceutical versus non-pharmaceutical. Most studies looked at research at a high level of aggregation.

#### Countries studied

The majority of the studies that analysed economies of scale (33/51) took an individual country as the focus of analysis and most of the rest studied two or more countries internationally. There were few purely sub-national analyses – six of the papers focused on local level analyses of individual research institutes or departments and it was at this level that diseconomies of scale were most likely to be identified (3/6), whereas positive economies of scale were found just once, constant returns to scale were found once and no clear result was found once. Kretschmer [32] analysed research activity in molecular biology by some 450 scientists in 56 research groups, but the source of data and the country to which the analysis refers are not clearly stated in the paper.

Most of the papers we reviewed in detail looked at research in one or more countries in Europe (24/51) or North America (16/51) or both (3/51). The remaining eight studies were either multinational beyond those regions or concerned individual countries in other regions (e.g. Taiwan), with the exception of Kretschmer [32], in which it was not possible to identify the country conclusively. Once we exclude those articles whose results indicate both economies and diseconomies of scale, the remaining 43 articles show a pattern of a slightly higher probability of finding economies of scale than diseconomies of scale regardless of geography.

#### Summary of findings about economies of scale

In summary, the empirical literature is varied both in method and findings, but taken as a whole, implies a greater likelihood of finding positive economies of scale in biomedical and health research than diseconomies, or constant returns to scale, although all three kinds of findings are common. This literature is largely focused at the whole-university or whole-institute level and usually takes a national perspective. Results differ across the wide range of institutions analysed as well as the outputs selected. Taken together, the literature does not permit conclusions to be drawn about different kinds of research within the overall heading of biomedical and health research.

### Economies of scope in biomedical and health research

Having discussed economies of scale we now turn to economies of scope. We found 22 papers that presented evidence on (dis-)economies of scope in biomedical and health research. Nearly half of these studies (10/22) found evidence of positive economies of scope. In other words, they found that the total cost of producing multiple research outputs in one place would be lower than the total cost of producing the same volume and mix of outputs in multiple places with each place only concentrating on a single type of output. However, five of the 22 studies found evidence of diseconomies of scope – implying that it would be more efficient to concentrate research in any one place on a narrower range. The remaining seven studies found evidence of both economies and diseconomies of scope in research (Table 4).

**Table 4:** Numbers of papers finding (dis-)economies of scope in biomedical and health research – by type of research group whose productivity is being analysed

	Research Team^a^	Department^b^	University/Research Institute	Total
Diseconomies of scope	–	1	4	5
Economies of scope	2	1	7	10
Evidence for both	–	1	6	7
Total	2	3	17	22

^a^Research Team, Sub-departmental laboratory/research unit

^b^University department or research centre inside a bigger organisation. A department is typically composed of a number of research teams

Studies of economies of scope were most commonly focused at the level of whole universities or research institutes (17/22) (Table 4). Seven of the 17 papers analysing at this level found positive economies of scope in research, but four found diseconomies of scope and the other six showed evidence for both economies and diseconomies. Thus, overall, there is no dominant picture emerging at this level of research group. There were only three studies focused at the level of departments within universities and institutes, and taken together they present mixed results – one study finding economies of scope [52], one finding diseconomies [53] and one finding evidence for both [54]. The two studies focused at the ‘Research team’ level support economies of scope at that level.

Twenty of the 22 studies looking at scope had also assessed economies of scale. Consequently, it is no surprise that the same range of measures of research outputs was evident in studies of scope as in studies of scale. However, when scope was analysed, it was less common for publications to have been used to measure research output. Whereas half of the papers analysing economies of scale (26/51) had included publications as an output, a slightly smaller proportion (9/22) of papers looking at economies of scope did so, two of which considered citations. Contrastingly, 15 of the studies that included scope in their analysis used postgraduate and undergraduate student numbers, separately, as outputs. Nine of the times when student numbers were among the outputs (and not on any other occasions), the amount of research funding raised was also included as an output, on the basis that more productive research groups will attract more funding. Postgraduate student numbers, specifically PhDs and other research-based degrees, might justifiably be seen as research (plus teaching) outputs, though undergraduate student numbers are a proxy for teaching output alone. However, PhD students are also an input to research, as well as a partial proxy for output. Grants and other external funding received are inputs to biomedical and health research as well as outputs from it, so the direction of causation runs both ways.

Although they are crude measures, and are far from representing the full fruits of biomedical and health research, counts of publications and patents are at least outputs from research, and are arguably even better proxies if quality-adjusted in some way. Nine of the 22 studies of economies of scope used publications as their measure of research output, and two of those also used patents (no study used patents without also using publications as a research output). The two studies using both publications and patents as outputs were those of Chavas et al. [21] and Foltz et al. [41], both of which are of United States universities and found significant economies of scope in producing publications and patents. Foltz et al. [41] estimated two regressions, one using only quantitative output measures and another adjusting the quantity of articles and patents according to their quality by considering the number of citations. Significant evidence of economies of scope was found only for the case in which the outputs where adjusted by the number of citations.

The nine studies where publications, alone or with patents, were used as a measure of research output were more likely than the others to show the presence of positive economies of scope – six of the nine did so (including both papers that also used patents as a research output), compared with two showing diseconomies of scope, and one showing evidence of both economies and diseconomies of scope.

Of the 13 (out of 22) studies of scope that did not use publications or patents to measure output, two used instead the quality of research as measured by the United Kingdom Research Assessment Exercise and one [53] used another quality index. Most of the remainder used postgraduate (and, separately, undergraduate) student numbers, usually in combination with a measure of research funding. Undergraduate student numbers were used as a proxy for teaching output in studies of universities as multi-product firms with the products ‘teaching’ and ‘research’. Postgraduate student numbers were used in a similar way as a proxy for graduate teaching, where that was distinguished from undergraduate teaching. However, as discussed earlier, PhD students can be seen as an input to research activity as well as a proxy for the output of graduate teaching. This makes those findings problematic to interpret, e.g. a research group with more PhD students that produces more publications is not necessarily being more productive per unit of research labour if PhD students are considered in part to contribute to the research labour force.

#### Results by type of model used

All three of the types of econometric methods described earlier were once again in evidence. Six of the 22 papers concerned with scope used a proxy variables approach, 11 used the cost function method, and five used production functions (Table 5). Overall, the studies were more likely to find positive economies of scope (10/22) than diseconomies of scope (5/22), with seven studies finding evidence of both economies and diseconomies.

**Table 5:** Numbers of papers finding (dis-)economies of scope in biomedical and health research

	Proxy variables	Multi-product cost function	Multi-output production function	Total
Diseconomies of scope	1	3	1	5
Economies of scope	4	5	1	10
Evidence for both	1	3	3	7
Total	6	11	5	22

#### Types of biomedical and health research

The likelihood of economies or diseconomies of scope might be expected to differ according to the particular types of biomedical and health research that are being conducted. However, the studies we found in the literature are mostly at too high a level of aggregation to permit distinct conclusions to be drawn about the presence of economies or diseconomies of scope for different sub-headings of research within ‘biomedical and health’, such as basic research versus clinical research, or pharmaceutical versus non-pharmaceutical.

#### Countries studied

As in the case of the literature about economies of scale, the settings for the papers considering economies of scope were mainly Europe (12/22), North America (8/22) or both (1/22), with just one having a setting elsewhere, namely in Bangladesh [19]. While in Europe the number of articles suggesting economies and diseconomies of scope is the same, in the case of North America no article suggests purely diseconomies of scope. Five of the articles with a focus in North America indicate positive economies of scope and three show evidence for both economies and diseconomies of scope. The focus of studies looking at scope was never at a local, sub-national level.

As mentioned above, only two studies analysed solely economies of scope without mentioning economies of scale. Cherchye et al. [54] assumed that a multi-output production process is closely related with the concept of economies of scope, since the process depends on the joint use of input to produce a set of outputs. Using a non-parametric characterisation of cost-efficient behaviour, their results suggest that, while the best universities perform well in all areas in which they are involved, less efficient universities can also perform very well in their fields of specialisation. In the case of Glass et al. [55], they compare more- and less-specialised universities to explore which system yields relatively higher performance. Through the use of non-parametric DEA-based models incorporating financial ratios, the findings indicate that more-specialised university production results in better performance on average than less-specialised production.

#### Summary of findings about economies of scope

The effect on research outputs of varying the scope of activities by a research group was less often reported in empirical studies than the effect of scale. Taken overall, the empirical literature more often suggests the existence of positive economies of scope in research than diseconomies, but the picture is mixed, perhaps owing to the variety of settings focused on, and the methods used, in the quantitative analyses. The use of postgraduate student numbers and/or external research funding won is problematic as they are inputs to the research process as well as partial proxies for research outputs. Economies of scope were predominantly tested at the whole university/institute level.

## Conclusions

Evidence on the presence of economies or diseconomies of scale or scope when undertaking biomedical and health research in publicly funded institutions is scattered around a variety of literatures. We were able to identify 60 papers up to November 2014 that presented relevant evidence. A wide variety of analytical approaches were adopted, in an even wider variety of settings. It is impractical in a single article to represent the full diversity of the literature, but some overall conclusions do emerge. The large majority of econometric analyses relied on one or another of three main types of model for investigating economies of scale and/or scope, respectively using either proxy variables or multi-product cost functions or multi-product production functions. The type of model used did not noticeably affect the conclusions – all three types of approach found, in different papers, positive economies of scale, or diseconomies, or evidence for both, and likewise for economies of scope.

### Challenges of measurement

There is no perfect measure for research outputs. The most commonly used measures concern numbers of publications and/or citations. A small number of studies have referred to numbers of patents. A measure of ‘output’ commonly found was student numbers, separately identifying postgraduate and undergraduate students. PhDs are arguably a proxy for part of the research output of institutions of higher education and other publicly funded institutes, but they are also an input to that research, which makes interpreting the findings of econometric studies that included them problematic. Undergraduate student numbers are a proxy for teaching output in studies that view institutions of higher education as multi-product firms producing research, postgraduate teaching and undergraduate teaching. Several studies included levels of external research funding (e.g. grants won) as a proxy for research output, but this makes interpretation problematic as research funding is necessarily strongly correlated with the cost and quantity of the inputs to research.

### Effects of scale and scope

The level of aggregation of the ‘research group’ being studied affects the balance of likely conclusions about the existence or otherwise of economies of scale. Where the empirical evidence is clearest is at the level of universities or research institutes, where studies more often point to the existence of positive economies of scale than to diseconomies of scale in biomedical and health research. However, both findings exist in the literature, along with findings of inverse U-shaped relationships which imply that, although economies of scale exist at modest size ranges for organisations undertaking publicly funded research, they eventually transform into diseconomies as scale increases further.

At more disaggregated organisational levels, particularly at the level of individual research units, laboratories or projects, the numbers of studies are smaller and evidence is very mixed. According to the particular study, economies of scale are found, or diseconomies, or neither, or both. There is too little evidence to identify where the turning points lie in those cases, and they vary significantly according to the particular type of research undertaken, but they are expressed in tens of staff rather than hundreds.

Economies of scope have been less frequently studied in the context of biomedical and health research than have economies of scale. Overall, the literature more often suggests the existence of positive economies of scope than diseconomies; implying that organisations undertaking a variety of research will be more productive than a collection of separate organisations each pursuing only one of those research areas, but the picture is again mixed.

We found insufficient evidence to discern different degrees of (dis-)economies of scale or scope for different types of biomedical and health research or different geographical regions.

The absence of predominant findings for or against the existence of economies of scale or scope implies a continuing need for case by case decisions when distributing research funding, rather than a general policy either to concentrate funding in a few centres or to disperse it across many.

We acknowledge a number of limitations of the study. First, it was not feasible to compare the quality of the selected articles. We tested the feasibility of formally and systematically evaluating the papers by drawing on and adapting two existing frameworks as suggested by Gough [5]. However, the wide variety of methodologies and approaches found in the literature hinder comparison of the quality of the articles. Therefore, it is recommended for future studies to develop a publication quality tool that reflects the main characteristics that an article related to economies of scope and/or scale should have.

Second, REA does not cover all available literature, and could result in evidence being missed from the analysis. However, after considering the relative costs and benefits of REA and Systematic Literature Review, we selected REA as the more appropriate method for the current analysis. Moreover, the limitation related to the use of the REA is partially compensated by the fact that (1) the search was done using a long list of databases (EconLit, ASC, BSC, Cinhal, PubMed, Scopus, and Web of Science), (2) it was supported by a posterior ‘snowballing’ of the references included in the papers identified, and (3) grey literature was included.

Third, given the scope of the project, we did not explore economies of scale and scope in private sector R&D, where internal and external synergies are also taking place. Therefore, it is recommended for future studies to include economies of scale and scope related to the private sector.

Despite the limitations, this literature review is the first to be focused on the economies of scale and scope in publicly funded biomedical and health research, bringing light into a topic of high relevance for both funders and researchers. We found a large, disparate literature of relevance, containing studies of widely varying types in terms not just of methodology and whether investigating scale or scope or both, but also of the level of ‘research group’ investigated, types of research within and beyond ‘biomedical and health’, whether including teaching in the study alongside research, geographic location and vintage. What is clear is that there remains plenty of room for further research into where and how economies of scale or scope can be exploited and diseconomies avoided. In particular, more work is needed to understand how these economies or diseconomies play out at lower levels of aggregation (below the whole institute or university level) and to understand the role of economies or diseconomies of scope rather than scale. Furthermore, with public funding of research under pressure in most economies, the incentive to identify how best to organise biomedical and health research most effectively and efficiently is clear and strong.

## Abbreviations

DEA: data envelopment analysis
REA: rapid evidence assessment
